# MiRNAs as new potential biomarkers and therapeutic targets in brain metastasis

**DOI:** 10.1016/j.ncrna.2024.02.014

**Published:** 2024-02-23

**Authors:** Ozal Beylerli, Huaizhang Shi, Sema Begliarzade, Alina Shumadalova, Tatiana Ilyasova, Albert Sufianov

**Affiliations:** a Central Research Laboratory, Bashkir State Medical University, Ufa, Republic of Bashkortostan, 3 Lenin Street, 450008, Russia; b Department of Neurosurgery, The First Affiliated Hospital of Harbin Medical University, Youzheng Street 23, Nangang District, Harbin, Heilongjiang Province, 150001, China; c Department of Oncology, Radiology and Radiotherapy, Tyumen State Medical University, 54 Odesskaya Street, 625023, Tyumen, Russia; d Department of General Chemistry, Bashkir State Medical University, Ufa, Republic of Bashkortostan, 3 Lenin Street, 450008, Russia; e Department of Internal Diseases, Bashkir State Medical University, Ufa, Republic of Bashkortostan, 450008, Russia; f Department of Neurosurgery, Sechenov First Moscow State Medical University (Sechenov University), Moscow, 119992, Russia; g Educational and Scientific Institute of Neurosurgery, Рeoples’ Friendship University of Russia (RUDN University), 6 Miklukho-Maklaya St, Moscow, 117198, Russia

**Keywords:** Brain metastases, microRNA, Replacement therapy, Metastatic cascade, Biomarkers

## Abstract

Brain metastases represent a formidable challenge in cancer management, impacting a significant number of patients and contributing significantly to cancer-related mortality. Conventional diagnostic methods frequently fall short, underscoring the imperative for non-invasive alternatives. Non-coding RNAs (ncRNAs), specifically microRNAs (miRNAs) and long non-coding RNAs (lncRNAs), present promising avenues for exploration. These ncRNAs exert influence over the prognosis and treatment resistance of brain metastases, offering valuable insights into underlying mechanisms and potential therapeutic targets. Dysregulated ncRNAs have been identified in brain metastases originating from various primary cancers, unveiling opportunities for intervention and prevention. The analysis of ncRNA expression in bodily fluids, such as serum and cerebrospinal fluid, provides a noninvasive means to differentiate brain metastases from primary tumors. NcRNAs, particularly miRNAs, assume a pivotal role in orchestrating the immune response within the brain microenvironment. MiRNAs exhibit promise in diagnosing brain metastases, effectively distinguishing between normal and cancer cells, and pinpointing the tissue of origin for metastatic brain tumors. The manipulation of miRNAs holds substantial potential in cancer treatment, offering the prospect of reducing toxicity and enhancing efficacy. Given the limited treatment options and the formidable threat of brain metastases in cancer patients, non-coding RNAs, especially miRNAs, emerge as beacons of hope, serving as both diagnostic tools and therapeutic targets. Further clinical studies are imperative to validate the specificity and sensitivity of ncRNAs, potentially reshaping approaches to tackle this challenge and elevate treatment outcomes for affected patients.

## 1 Introduction

**Fig. 1:**
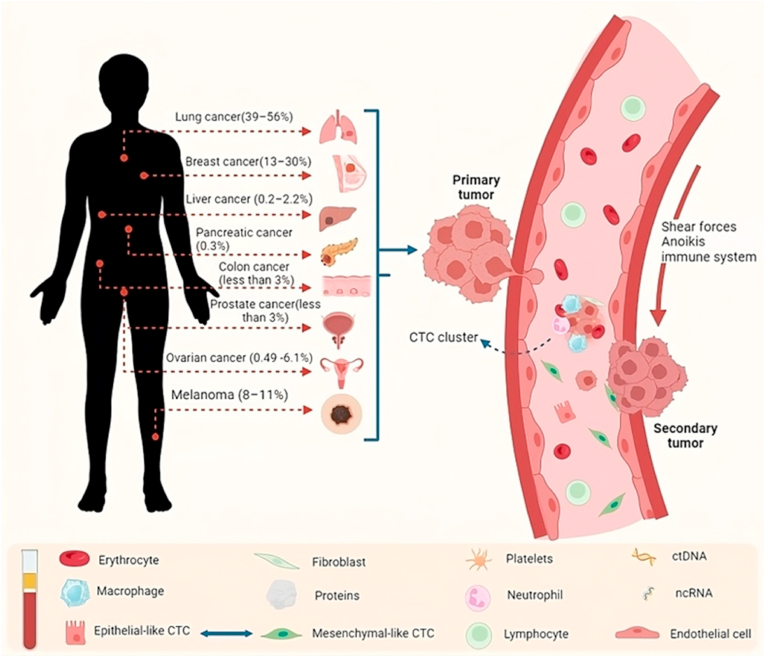
Illustrates the distribution pattern of brain metastases, highlighting that metastatic lesions commonly target the brain originating from lung cancer, breast cancer, melanoma, and, to a lesser extent, cancers originating in the gastrointestinal tract, prostate, ovaries, and other sources.

Metastatic brain tumors (MBTs) are secondary tumors originating in other parts of the body, eventually spreading to the brain either through the bloodstream or direct tissue invasion [1]. They are the most prevalent type of brain tumors, occurring in around 25% of all metastatic cancers and affecting approximately 20–40% of adult cancer patients. Without intervention, survival is typically only 1–2 months, slightly improving to around 6 months with treatment. The challenge lies in the fact that over half of MBT patients have multiple tumors, reducing the effectiveness of localized treatments [2]. The rising incidence of MBTs may be attributed to improved imaging, better primary tumor treatments, and increased patient survival (Fig. 1).

Various approaches have been employed to understand the mechanisms of brain metastasis, with a growing focus on the potential role of microRNAs in the diagnosis and treatment of primary and metastatic brain tumors [[3], [4], [5]]. MicroRNAs, small non-coding RNAs 19–25 nucleotides in length, play a crucial role in biological regulation. Naturally produced in cells by cleaving larger RNA strands, there are currently 1048 known human microRNAs that modulate about 3% of all genes and up to 30% of protein-coding genes [6]. Their function involves binding to complementary sites on the 3′ untranslated region (UTR) of genes, leading to the recruitment of protein complexes that hinder translation and/or decrease mRNA stability. MicroRNAs are vital for protein expression and are closely linked to both normal and abnormal biological processes [[7], [8], [9], [10], [11]]. The altered expression of microRNAs in cancer cells compared to normal cells has sparked interest in using microRNA profiling to identify cancer cell origin and assess therapy response.

This review explores the function of microRNAs, their potential applications in diagnosis and prediction brain metastasis, and the controlled expression of microRNAs as a strategy for treatment MBTs. The discussion also includes the prospects of treating MBTs through innovative therapies target microRNAs.

## 2 Dysregulated mirnas in brain metastases

NcRNAs occupy a central role in governing the human genome, and their aberrant expression significantly contributes to the initiation of tumors, the metastatic process, and resistance to treatment [12]. Despite this, the precise role of ncRNAs in the context of brain metastases and their involvement in the development of therapy resistance has remained obscured. MicroRNAs, a subset of naturally occurring small non-coding RNAs, function through post-transcriptional control, negatively regulating gene expression by binding to the 3′-untranslated region (3′-UTR) of their target mRNAs [13]. Changes in microRNA profiles have profound consequences, leading to modified protein expression and a cascade of downstream effects, including oncogenesis. MicroRNAs implicated in oncogenesis are referred to as OncomiRs [14]. Typically, decreased levels of microRNAs are associated with tumor-suppressive effects, while increased expression is linked to oncogenic effects, although this pattern may not be consistent across all cases [15]. The role of miRNAs in cancer development encompasses various aspects, including alterations in the cell cycle, proliferation, metastasis, invasion, and angiogenesis.

In the context of metastatic brain tumors (MBTs), microRNAs appear to play a crucial role in the process of metastasis to the brain [16]. Understanding the regulation of microRNAs is essential for targeting them in diagnostic and therapeutic applications. MicroRNAs play central roles in processes related to cell development, proliferation, differentiation, and apoptosis. The loss of tumor-suppressive miRNAs can activate intrinsically oncogenic pathways, leading to cancer-related characteristics, tumor initiation, progression, and metastasis [13].

In a recent investigation by Okuno et al. [17], they evaluated a novel microRNA-based qRT-PCR test utilizing microRNA biomarkers to discern the tissue of origin for metastatic brain tumors (MBTs). The test demonstrated a sensitivity exceeding 90% and a specificity ranging from 97% to 100%. In a patient cohort characterized by brain carcinomas with unidentified primary tumors, this test accurately diagnosed approximately 80% of the cases. Certain specific microRNAs, such as miR-10b, miR-21, and miR-200 - particularly elevated in the cerebrospinal fluid (CSF) of MBT patients—have been associated with MBTs. This high level of accuracy could prove exceptionally valuable in clinical settings.

Another significant study highlighted that, beyond identifying the tissue of origin for MBTs, microRNA profiling can distinguish primary from secondary brain tumors. Notably, hsa-miR-92b and hsa-miR-9/hsa-miR-9* were found to be overexpressed in primary tumors compared to secondary brain tumors. This discrimination can be achieved with a sensitivity of 88% and a specificity of 100% [18]. The findings from these studies underscore the potential of microRNA-based diagnostics in enhancing both the accuracy of MBT diagnosis and the differentiation between primary and secondary brain tumors. These advancements represent a promising avenue for improving clinical outcomes and refining treatment strategies for patients with metastatic brain tumors.

In recent findings, numerous ncRNAs have been identified as being dysregulated in brain metastases when compared to primary tumors. Specifically, Xiong et al. observed upregulation of microRNA-92b, microRNA-9, and microRNA-9* in primary brain tumors compared to brain metastases, indicating the potential use of these microRNAs as biomarkers to distinguish between the two types of tumors [19]. Another study conducted by Ji et al. revealed the association of several dysregulated non-protein-coding genes with brain metastases [20]. Among these genes, MIR124-2, RP11–713P17.4, and NUS1P3 were found to be hypermethylated, while CTD-2023M8.1, MIR3193, and MTND6P4 were hypomethylated in brain metastases compared to primary brain tumors. These findings suggest that the epigenetic regulation of genes encoding ncRNAs can impact their expression, leading to altered levels and influencing tumor cell invasion, proliferation, and migration. The role of miRNAs in the biology of brain metastases has been unequivocally demonstrated through investigations involving various primary tumor types (Table 1) [[21], [22], [23], [24], [25], [26], [27], [28], [29], [30], [31], [32], [33], [34], [35], [36]]. This expanding body of research heralds the promising role of miRNAs in unraveling the complexities of cancer and improving diagnostic and therapeutic approaches.

**Table 1:** provides a summary of the modified miRNA profiles observed in brain metastases in comparison to their primary tumor counterparts. These changes in miRNA expression have been identified in various scenarios, such as non-small cell lung cancer (NSCLC), where certain miRNAs are associated with factors like matrix metalloproteinases (MMPs), vascular endothelial growth factor (VEGF), protein tyrosine phosphatase-1B (PTB1b), and hypoxia-inducible factor 1-α (HIF-1α).

miRNAs	Regulation	Primary tumor	Putative target	References
**miR-19a**	Down	Breast	The 3′-untranslated region (3′-UTR) of the tissue factor transcript	[21]
**miR-29c**	Down	Breast and melanoma	Induced the maturation of myeloid leukemia cells, leading to the production of the MCL1 protein	[22]
**miR-31**	Down	Colon	p53	[23]
**miR-200**	Up	Breast and lung	ZEB1 and ZEB2, functioning as transcriptional suppressors, impede the transcription of E-cadherin	[24]
**miR-210**	Up	Breast and melanoma	PTP1b and HIF-1α	[25]
**miR-1258**	Down	Breast	Heparanase	[26]
**miR-7**	Down	Breast	KLF4 gene	[27]
**miR-145**	Down	Lung adenocarcinoma	3′-UTR of the JAM-A and fascin	[28]
**miR-328**	Up	NSCLC	PRKCA gene	[29]
**miR-378**	Up	NSCLC	MMP-7, MMP-9 and VEGF	[30]
**miR-146-a**	Down	Breast	B-catenin and hnRNPC	[31]
**miR-768-3p**	Down	Lung and breast	K-RAS	[33]
**miR-1, miR-145, miR-146a, miR-143, miR-10b, miR-22**	Up	Colon	Many genes are linked to both apoptosis (programmed cell death) and the progression of cancer	[36]

In the context of breast cancer, changes in miR-1258 have been directly associated with the expression of heparinase, a well-known pro-metastatic enzyme prevalent in brain metastatic breast cancer cells. Heparinase functions by breaking down heparan sulfate chains, influencing the cytoskeleton, and enhancing the cells' capacity to overcome the formidable blood-brain barrier (BBB) [26,27]. The migratory and invasive capabilities of breast cancer stem cells (CSCs) have been demonstrated to be closely linked to the expression of the KLF4 gene, showing an inversely proportional relationship with miR-7 expression [28]. Likewise, in the context of lung cancer, the diminished expression of miR-145 has been associated with the progression of lung adenocarcinoma and has facilitated the formation of brain metastases [29]. In non-small cell lung cancer (NSCLC), miR-328 has been involved in regulating cell migration and the occurrence of brain metastases by influencing the expression of the PRKCA genes [30]. In NSCLC, miR-378 has been demonstrated to enhance the development of brain metastases by modulating the expression levels of key genes associated with angiogenesis and extracellular matrix invasion, such as MMP-7, MMP-9, VEGF, and Sufu [31]. Notably, members of the miR-200 family have shown significant elevation in the CSF of patients with metastatic brain lesions originating from various primary tumor types. This contrasts with patients with glioblastoma and individuals without cancer [32]. This growing body of research highlights the crucial role of miRNAs in the complex processes underlying brain metastases and offers valuable insights into potential diagnostic and therapeutic approaches for effectively managing these challenging conditions.

The dynamic and central role of the brain microenvironment, primarily characterized by astrocytes, becomes evident in promoting increased growth and resistance to chemotherapy in metastatic brain tumors. Astrocytes stimulate the upregulation of various genes associated with the survival of neighboring tumor cells, rendering them more aggressive, irrespective of the primary tumor's histological origin or the presence of p-glycoprotein. Changes in microRNA profiles of tumor cells during their transition from the primary tumor to the brain microenvironment suggest that miRNAs play a direct role in changes caused by the brain microenvironment in metastatic tumor cells. For instance, rhabdoid tumor cells exhibit distinct miRNA profiles when originating in the brain compared to the kidney [33]. In animal models, the suppression of miR-146a was observed in brain metastases compared to the original tumors, and this was associated with reduced levels of β-catenin protein and increased expression of heterogeneous nuclear ribonucleoprotein C1/C2 (hnRNPC), potentially enhancing migratory and invasive properties [34]. MiR-768–3p undergoes downregulation in tumor cells during co-culture with astrocytes, a finding validated in human brain metastatic tissues from lung cancer, breast cancer, and melanoma, in comparison to match-paired primary tumors from the same patients. This downregulation of miR-768–3p leads to increased K-ras expression, resulting in heightened tumor growth and drug resistance [35]. Notable differences in miRNA profiles were identified between primary colorectal tumors and matched metastatic brain tumors [36]. Brain-metastatic carcinomas exhibited the overexpression of miRNAs, such as miRNA-145, 1, 146a, 576–5p, 126*, HS287, 28–5p, 143, 199b-5p, 199a-5p, 10b, 22, 133b, 145*, 199a, 133a, 125b, alongside the downregulation of miR-31 and HS170. Additionally, miRNAs isolated from exosomes of parental breast cancer and melanoma cells showed variations from those isolated from their corresponding metastatic brain variants. Brain metastases exhibited overexpression of miRNA-210 and downregulation of miRNAs-19a and 29c [35]. Taken together, these studies illustrate that the brain microenvironment induces alterations in the miRNA signature of tumor cells, activating pro-growth signaling pathways and resulting in more aggressive, drug-resistant metastatic lesions. There is evidence suggesting that the influence of the microenvironment on tumor cells that "seed" in the brain may have a universal effect [32]. This concept provides a promising avenue to target key miRNAs involved in metastasis, potentially offering novel therapeutic strategies to manage this challenging condition. The intricate interplay between the brain microenvironment and miRNA expression underscores the need for further exploration in developing targeted interventions for metastatic brain tumors.

## 3 MIRNAS as therapeutics

**Fig. 2:**
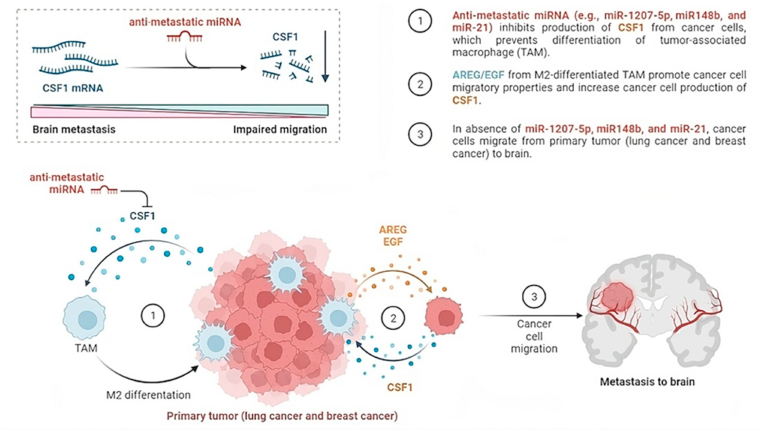
Illustrates that microRNAs (miRNAs) possessing anti-metastatic properties impede the occurrence of brain metastasis by target Colony Stimulating Factor 1 (CSF1). Tumor epithelium in various human epithelial cancers, including lung and breast cancer, expresses CSF1, which plays multifaceted roles in tumor progression and the metastatic process. Recent research has identified CSF1 as a novel target gene for miR-1207–5p, miR-148b, and miR-21, shedding light on its involvement in the metastasis of lung and breast cancers, which are primary sources of brain tumor metastases.

**Fig. 3:**
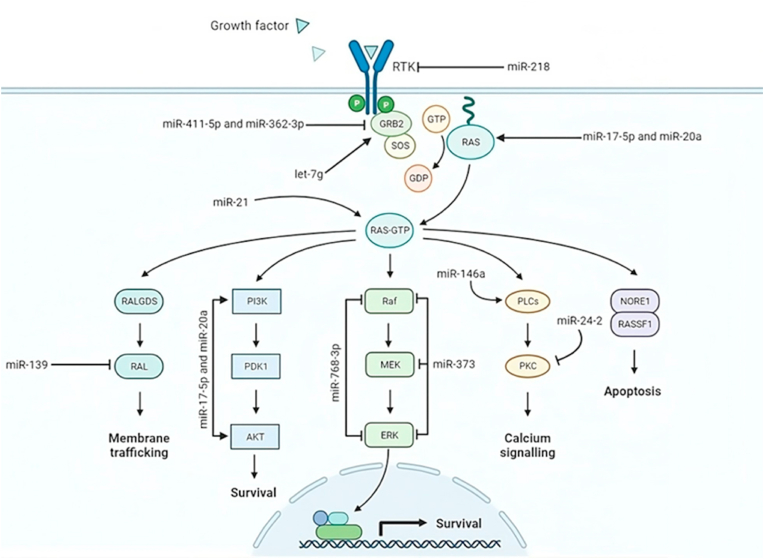
visually outlines the RAS/microRNAs (miRNAs) pathway in cancer metastasis, emphasizing the pivotal role of the Ras signaling pathway, particularly in tumor initiation and metastasis. In specific cancer types such as lung and breast cancer, the control of oncogenic RAS may be influenced by miRNAs. This is noteworthy since these cancers frequently display simultaneous dysregulation of RAS signaling and altered miRNA activity. MiRNAs function as crucial participants, either as oncogenes or tumor suppressors, guiding the progression of cancer and the intricate processes involved in metastasis, including the dissemination from primary tumors to the brain.

Advances in our understanding of miRNA biology and its intricate association with cancer development across various tumor types have prompted efforts to leverage this knowledge for miRNA-based therapeutics [37,38]. It is crucial to emphasize that there is presently a noticeable deficiency in the development of miRNA-based treatments specifically designed to address brain metastases. A well-established characteristic of miRNAs is their ability to regulate multiple genes, rendering endogenous miRNAs attractive targets for therapeutic interventions (see Fig. 2, Fig. 3).

Minn et al. investigated the molecular mechanisms underlying brain metastases in gastric cancer [39]. Despite the relatively low incidence of brain metastases in gastric cancer, the prevalence may increase with improved therapy and longer tumor survival. To better understand this phenomenon, their study focused on miRNA expression levels in combined primary gastric adenocarcinoma and metastatic brain adenocarcinoma. Using an Illumina miRNA microarray, the researchers identified differences in miRNA expression in eight cases. They found six upregulated miRNAs, and two downregulated miRNAs consistently present in all eight cases. Notably, two of these miRNAs, hsa-miR-141–3p and hsa-miR-200b-3p, belong to the miR-200 family. Further studies using online miRNA databases showed that the ZEB2 gene is the main target of hsa-miR141–3p and hsa-miR-200b-3p [39]. This discovery prompted researchers to focus on ZEB2 expression in metastatic brain adenocarcinoma. In support of their hypothesis, they observed a significant decrease in ZEB2 expression in some metastatic brain samples. The study also extended its investigation to metastatic gastric adenocarcinoma cell lines generated by in vivo selection following intracardiac injection of gastric cancer cell lines. Western blot analysis confirmed decreased ZEB2 expression in these metastatic cell lines. This work provides evidence that the expression of miR-200 family members, particularly hsa-miR-141–3p and hsa-miR-200b-3p, as well as their target gene ZEB2, is associated with brain metastasis of gastric adenocarcinoma [39]. This association is observed not only in matched patient samples but also in metastatic cell lines derived from in vivo selection, suggesting a potential role for these molecular factors in the development of brain metastasis in gastric cancer.

Ahmad et al. investigated the role of miR-10b in the development of brain metastases from primary breast cancer, a condition known for its difficult treatment and poor prognosis [40]. The results showed increased expression of miR-10b in primary breast cancer samples from patients who later developed brain metastases compared to those who did not. In addition, in vitro experiments showed that miR-10b increases the invasive potential of breast cancer cells. Statistical analysis using Wilcoxon signed-rank test revealed a significant difference in miR-10b expression between paired breast cancer tumors and brain metastases (p < 0.001). The study concluded that increased miR-10b expression is associated with breast cancer brain metastasis. The clinical significance of these findings is highlighted, suggesting that miR-10b could potentially serve as a prognostic and/or therapeutic target for antimetastatic therapy. Debeb et al. addressed the serious problem of brain metastasis in breast cancer with the goal of better understanding the biological and molecular basis of this process [41]. Researchers created new mouse models of brain metastasis by injecting green fluorescent protein (GFP)-tagged breast cancer cells into SCID/Beige mice via the tail vein. The study focused on the role of miR-141 in brain metastasis. Using knockdown and overexpression techniques with lentiviral vectors, the researchers observed that knockdown of miR-141 inhibited metastatic colonization of the brain, while ectopic expression of miR-141 enhanced metastatic colonization of the brain. The study achieved clinical relevance by measuring serum miR-141 levels in breast cancer patients (n = 105). The analysis showed that high serum levels of miR-141 were associated with shorter brain metastasis-free survival and served as an independent predictor of progression-free survival and overall survival. This study suggests that miR-141 plays a regulatory role in breast cancer brain metastasis. It proposes miR-141 as a potential biomarker and target for the prevention and treatment of brain metastases in breast cancer [41]. Gravgaard et al. performed global miRNA expression profiling on 47 tumor samples from 14 patients, including paired samples from primary breast tumors, matched lymph nodes, and distant metastases [42]. For this analysis, they used LNA-amplified miRNA microarrays. The study identified changes in microRNA expression associated with different stages of breast cancer progression. Key findings include the identification of 15 microRNAs whose expression differed significantly between primary tumors and corresponding distant metastases. Notably, miR-9, miR-219–5p, and miR-200 family members (involved in epithelial-mesenchymal transition) were among the differentially expressed miRNAs. Patients whose miRNA profiles clustered between the primary tumor and distant metastases had significantly shorter intervals between diagnosis and distant metastases compared to those whose profiles did not cluster. The study suggests a direct involvement of the miR-200 and miR-9 family in the metastatic process of breast cancer [42]. Harati et al. identified miR-101–3p as a critical miRNA that can significantly reduce breast cancer cell transmigration across the brain endothelium, a key event in the development of brain metastases [43]. The researchers observed that miR-101–3p expression was reduced in brain metastatic breast cancer cells compared to less invasive variants. In addition, miR-101–3p expression is inversely associated with the propensity of breast cancer cells to metastasize to the brain. Using a loss-gain-of-function approach, the study demonstrated that reducing miR-101–3p expression increased breast cancer cell transmigration across the brain endothelium in vitro. This effect was explained by the induction of COX-2 (cyclooxygenase-2) expression in cancer cells. Conversely, restoring miR-101–3p expression had the effect of reducing metastasis. Further experiments showed that miR-101–3p mediates its effects by modulating COX-2-MMP1 (matrix metalloproteinase-1) signaling. This signaling pathway is capable of disrupting interendothelial junctions, particularly claudin-5 and VE-cadherin, which are key components of the brain endothelium. The study concluded that miR-101–3p plays a critical role in the transmigration of breast cancer cells across brain endothelium by regulating the COX-2-MMP1 signaling pathway. The results indicate that miR-101–3p may serve as a therapeutic target to prevent or suppress brain metastasis in human breast cancer [43]. Giannoudis et al. analyzed differentially expressed miRNAs using primary breast cancer that did not recur (BCNR, n = 12), primary cancer that relapsed (BCR), and their paired brain metastases (BM, n = 40 pairs) using expression microRNA NanoString™ nCounter™ [44]. Analyzes. Microarray significance analysis identified 58 and 11 differentially expressed miRNAs between BCNR and BCR and BCR and BM, respectively. Pathway analysis revealed enrichment of genes involved in invasion and metastasis. Four miRNAs - miR-132–3p, miR-199a-5p, miR-150–5p, and miR-155–5p - were differentially expressed in both cohorts (BCNR-BCR, BCR-BM). Receiver operating characteristic curve analysis and the Kaplan-Meier survival method demonstrated their potential use as prognostic markers, showing significance in brain metastasis-free survival and overall survival. Ingenuity pathway enrichment linked these miRNAs to the MET oncogene. cMET protein was found to be overexpressed in BCR and BM cases compared to BCNR. The study suggests that this panel of 4-miRNAs could potentially distinguish breast cancer patients at increased risk of developing BCBM, offering insight into new therapeutic targets. In addition, target cMET is proposed for further study in the treatment of BCBM. Harati et al. investigated the molecular mechanisms underlying brain metastasis (BM) in breast cancer (BC), focusing on the role of miR-202–3p and its interaction with metalloproteinase-1 (MMP-1) [45]. The researchers used a variant of the human BC cell line MDA-MB-231 (MDA-MB-231-BrM2), selected for its propensity to form brain metastases, to examine the expression levels of MMP-1 and miR-202–3p. They used a gain-and-loss-of-function approach to modulate miR-202–3p levels and examined the effect on MMP-1 expression. The study also assessed the effect of miR-202–3p modulation on brain endothelial integrity and transmigration capacity of BC cells. The results showed that loss of miR-202–3p in breast cancer cells enhances their transmigration across the brain endothelium through upregulation of MMP-1. This activation disrupted interendothelial junctions including claudin-5, ZO-1, and β-catenin. Restoring miR-202–3p had a metastasis-suppressive effect and preserved the integrity of the endothelial barrier. This study identifies miR-202–3p as a critical regulator of brain metastasis and highlights the miR-202–3p/MMP-1 axis as a potential prognostic and therapeutic target. Roskova et al. analyzed miRNA expression in 71 fresh frozen histopathologically confirmed BM tissues originating from five common cancer types (lung, breast, renal cell carcinoma, colorectal carcinoma, and melanoma) [46]. MiRNA sequencing and subsequent validation by RT-qPCR were used to identify 373 miRNAs with significantly different expression across the five BM groups. A diagnostic classifier model was then developed based on the expression of six specific miRNAs (hsa-miR-141–3p, hsa-miR-141–5p, hsa-miR-146a-5p, hsa-miR-194–5p, hsa-miR- 146a-5p, hsa-miR-194–5p, hsa-miR-141–5p miR-200b-3p and hsa-miR-365b-5p). This classifier demonstrated an impressive ability to correctly classify 91.5% of samples. The study highlights the importance of studying dysregulated miRNA expression in BM and highlights the diagnostic potential of a validated 6-miRNA signature. Li et al. performed microRNA expression profiling in primary colorectal cancer and metastatic brain cancer and confirmed the results using quantitative real-time reverse transcription-polymerase chain reaction (RT-PCR) [47]. The results revealed the overexpression of several miRNAs, including miR-145, miR-1, miR-146a, miR-576–5p, miR-126*, HS287, miR-28–5p, miR-143, miR-199b-5p, miR- 199a-5p, miR-10b, miR-22, miR-133b, miR-145*, miR-199a, miR-133a and miR-125b in metastatic brain cancer. In addition, a decrease in the expression of miR-31 and HS170 was observed. Quantitative RT-PCR experiments with miR-125b confirmed the expression patterns found in the microarray experiments. The study concluded that miRNAs are differentially expressed in colorectal cancer and metastatic brain cancer. Ahmad et al. investigated the role of miR-20b in breast cancer brain metastasis, an aspect for which there are no well-established molecular biomarkers [48]. The study found that miR-20b expression was significantly higher in brain metastases of breast cancer patients compared with primary breast tumors and patients without brain metastases. To further explore the functional aspects, the researchers tested the effect of miR-20b overexpression on colony formation and invasion in vitro using MCF-7 and MDA-MB-231 cells. The results showed that miR-20b significantly induced both colony formation and invasiveness of breast cancer cells. Moreover, the study found increased levels of miR-20b in cells metastasizing to the brain compared to cells metastasizing to bone. These findings suggest a novel role for miR-20b in breast cancer brain metastasis [48]. The potential of miR-20b as a prognostic and/or therapeutic target is proposed.

Sereno et al. used a mouse model of BCBM and next generation sequencing (NGS) to identify changes in circulating microRNAs during the formation and progression of brain metastases [49]. Bioinformatics analysis was then performed to identify the corresponding targets of these miRNAs, and the results were further validated using samples of human brain metastases that had been resected from breast cancer patients. The study concluded that downregulation of circulating miR-802–5p and miR-194–5p is an early event in BCMC, and MEF2C emerges as a novel player in the development of brain metastases. Curtaz et al. identified two miRNAs that may serve as prognostic markers for brain metastases [50]. Specifically, the level of hsa-miR-576–3p was significantly increased and the level of hsa-miR-130a-3p was significantly decreased in exosomes from breast cancer patients with brain metastases. The area under the receiver operating characteristic curve (AUC) was used to evaluate diagnostic accuracy: AUC values were 0.705 and 0.699 for hsa-miR-576–3p and hsa-miR-130a-3p, respectively. In addition, correlation analysis revealed an association between microRNA levels and tumor markers. Hsa-miR-340–5p levels were significantly correlated with the percentage of Ki67-positive tumor cells, whereas hsa-miR-342–3p levels were inversely correlated with tumor stage [50]. This study suggests that analysis of miRNA expression levels in serum exosomes from breast cancer patients may identify non-invasive blood-borne prognostic molecular markers to predict the potential for brain metastasis in breast cancer. Dong et al. studied the roles of miR-21 in predicting the development of brain metastases (BM) from non-small cell lung cancer (NSCLC) [51]. A total of 132 patients with NSCLC were included, including 68 with BM and 64 without BM. NSCLC cells were collected and subjected to various assays to study the effect of miR-21 on cellular processes. The results showed that miR-21 expression was higher in NSCLC patients with BM compared to patients without BM. The study also showed that inhibition of miR-21 resulted in decreased expression levels and had significant effects on various cellular processes. Specifically, inhibition of miR-21 reduced cell proliferation, migration, invasion, and angiogenesis while promoting apoptosis in NSCLC cells. The study suggests that miR-21 may serve as a potential biomarker for prediction the development of brain metastases in patients with NSCLC. The observed effects on cellular processes further indicate a potential role for miR-21 in promoting aggressive behavior of NSCLC cells. Figueira et al. used an animal model of TNBC to analyze EVs in plasma through a nanoparticle tracking assay, confirming their origin across the blood-brain barrier (BBB) using flow cytometry [52]. Circulating miRNAs were assessed using quantitative reverse transcription-polymerase chain reaction (RT-qPCR), and their expression in the brain was assessed using in situ hybridization. A cellular model of BCBM formation combining TNBC cells and BBB endothelial cells has also been used to differentiate the origin of these biomarkers. The results showed that established brain metastases were associated with increased levels of circulating EVs, especially those of BBB origin. Notably, deregulation of miRNAs in the circulation was observed before the discovery of brain metastases, and their origin in the brain was suggested by correlating changes in the brain parenchyma. In vitro studies have shown that miR-194–5p and miR-205–5p are expressed and released by both breast cancer cells and endothelial cells during their interaction.

One of the newest meta-analyses aimed to thoroughly evaluate the diagnostic potential of circulating microRNAs in detecting brain tumors [53]. A total of 26 eligible studies were systematically reviewed to evaluate the diagnostic accuracy of miRNAs in BT. Key parameters such as sensitivity, specificity, positive likelihood ratio (PLR), negative likelihood ratio (NLR), diagnostic odds ratio (DOR), area under the curve (AUC), Q* index and summary receiver operating characteristic (SROC) carefully evaluated using Meta-Disc V.1.4 and Comprehensive Meta-Analysis V.3.3 software. The meta-analysis revealed high diagnostic accuracy of miRNAs in detecting BT: pooled sensitivity of 0.82 (95% CI: 0.816–0.84) and specificity of 0.82 (95% CI: 0.817–0.84). Additionally, the positive likelihood ratio (PLR) was 5.101 (95% CI: 3.99–6.51), the negative likelihood ratio (NLR) was 0.187 (95% CI: 0.149–0.236), and the diagnostic odds ratio (DOR) was 34.07 (95%CI: 22.56–51.43). The area under the curve (AUC) and Q* index further confirmed the high diagnostic performance of miRNAs with values of 0.92 and 0.86, respectively. The aim of this analysis was to reveal the diagnostic power of miRNAs in the detection of BT, providing insight into their performance in different contexts [53]. Thus, this comprehensive meta-analysis also suggests that circulating miRNAs hold promise as potential noninvasive markers for the early detection of brain tumors. The high diagnostic accuracy demonstrated across various parameters highlights the potential clinical utility of miRNA-based diagnostics in the field of neuro-oncology. Further research and validation are needed to confirm and extend these findings, paving the way for the development of reliable and accessible tools for the early diagnosis of brain tumors (Table 2).

**Table 2:** The significance of microRNAs (miRNAs) as valuable tools in cancer diagnosis and treatment.

Direction	Type of tissue/tumor	Type of miRNAs	Reference
**Diagnosis of tumor progression**	Breast cancer	Hsa-miR-141	[54]
	Breast cancer	Hsa-miR-17, Hsa-miR-502, Hsa-miR-532, Hsa-miR-500, Hsa-miR-362	[55]
	Hepatocellular carcinoma	Hsa-miR-141, Hsa-miR-126, Hsa-miR-200c	[56]
	Breast cancer	Hsa-miR-10b, Hsa-miR-21, Hsa-miR-200	[57]
**Application for therapy**	Metastatic breast cancer	Hsa-miR-335	[58]
	Metastatic melanoma	Hsa-miR-1258	[59]
	HepG2 cells	Hsa-miR-122	[60]
	Hepatocellular carcinoma	Let-7	[61]
	Hepatocellular carcinoma	Hsa-miR-122	[62]
	Breast cancer	Hsa-miR-145	[63]

## 4 Conclusions

Brain metastases are a frequent consequence of advanced cancer, significantly impacting patient well-being. The increased incidence is attributed to enhanced treatment efficacy and improved survival rates, allowing metastatic cells more time to breach the blood-brain barrier. Prediction and addressing this challenge necessitate innovative diagnostic biomarkers, particularly small RNA molecules like miRNAs. Dysregulated miRNA expression is evident across cancers, implicating their roles in the intricate process of metastasis. Identifying miRNAs specific to brain metastasis holds promise as predictive markers, potentially transforming personalized treatment approaches. Despite progress in miRNA-based therapeutics, challenges persist, including off-target effects and toxicity. Addressing these challenges requires precise dosing, reliable delivery systems, and efficient blood-brain barrier traversal. The prognosis for brain metastasis patients remains challenging due to the lack of effective treatment options. MiRNAs play pivotal roles in cancer, and their distinct profiles in brain metastases offer potential targets for intervention. Despite obstacles, ongoing research and advancements in miRNA diagnostics and therapeutics provide hope for improving outcomes in confronting this formidable clinical challenge.

## Funding

This work was supported by the Bashkir State Medical University Strategic Academic Leadership Program (PRIORITY-2030).

## CRediT authorship contribution statement

**Ozal Beylerli:** Supervision, Conceptualization. **Huaizhang Shi:** Conceptualization. **Sema Begliarzade:** Writing – original draft. **Alina Shumadalova:** Writing – original draft, Resources. **Tatiana Ilyasova:** Writing – original draft, Resources. **Albert Sufianov:** Writing – review & editing.

## Declaration of competing interest

Ozal Beylerli is an editorial board member for Non-coding RNA Research and was not involved in the editorial review or the decision to publish this article. All authors declare that there are no competing interests.
